# Diurnal Regulation and Gene-Specific Vulnerability of Oxidative Alcohol-Metabolizing Enzymes to Circadian Disruption

**DOI:** 10.3390/ijms27042041

**Published:** 2026-02-22

**Authors:** Yool Lee, Ali Keshavarzian, Byoung-Joon Song

**Affiliations:** 1 Department of Translational Medicine and Physiology, Elson S. Floyd College of Medicine, Washington State University, Spokane, WA 99202, USA; 2 Department of Integrative Physiology and Neuroscience, College of Veterinary Medicine, Washington State University, Pullman, WA 99164, USA; 3 Sleep and Performance Research Center, Washington State University, Spokane, WA 99202, USA; 4 Steve Gleason Institute for Neuroscience, Washington State University, Spokane, WA 99202, USA; 5 Rush Center for Integrated Microbiome and Chronobiology Research, Rush University Medical Center, Chicago, IL 60612, USA; ali_keshavarzian@rush.edu; 6 Department of Internal Medicine, Division of Digestive Diseases and Nutrition, Rush University Medical Center, Chicago, IL 60612, USA; 7 Departments of Physiology, Rush University Medical Center, Chicago, IL 60612, USA; 8 Department of Anatomy and Cell Biology, Rush University Medical Center, Chicago, IL 60612, USA; 9 Section of Molecular Pharmacology and Toxicology, National Institute on Alcohol Abuse and Alcoholism, Bethesda, MD 20892, USA; bj.song@nih.gov

**Keywords:** circadian rhythm, alcohol metabolism, ALDH2, CYP2E1, BMAL1, CLOCK, HNF4A, sleep deprivation, high-fat diet, shift work, pathologies

## Abstract

Oxidative alcohol metabolism in the liver relies on sequential enzymatic reactions involving alcohol dehydrogenase (ADH), cytochrome P450 2E1 (CYP2E1), and aldehyde dehydrogenase (ALDH) isozymes. However, the circadian regulation of these enzymes, their susceptibility to genetic, environmental, and metabolic disruption, and their functional implications toward alcohol-mediated tissue injury remain incompletely defined. To address this gap, we performed a comprehensive integrative analysis of the publicly available circadian transcriptome datasets spanning genetic clock disruption, acute sleep deprivation, chronic high-fat diet feeding, and occupational shift work to systematically characterize the temporal regulation and disruption vulnerability of the major alcohol-metabolizing enzymes. Mouse tissue-cycling analyses revealed pronounced gene- and tissue-specific diurnal regulation, with *Adh1* oscillating primarily in adipose tissues; *Cyp2e1* and mitochondrial *Aldh2* cycling broadly across kidney, aorta, lung, adrenal gland, and liver; and cytosolic *Aldh1b1* being uniformly arrhythmic. In the liver, *Cyp2e1* and *Aldh2* exhibited robust ~24 h oscillations that peaked during the light/resting phase, while *Adh1* showed inconsistent rhythmicity and *Aldh1b1* remained arrhythmic. Notably, *Cyp2e1* and *Aldh2* rhythms persisted in *Bmal1* knockout and *Clock* mutant livers under light–dark conditions, despite complete loss of core clock gene oscillations, yet were abolished in constant darkness, revealing that systemic zeitgeber cues can mask the loss of intrinsic clock function to maintain apparent rhythmicity in these metabolic genes. Systematic cross-paradigm comparison established a novel gene-specific vulnerability hierarchy. *Aldh2* was found to be most disrupted by environmental and metabolic perturbations, with acute sleep deprivation eliminating its rhythmicity and temporal expression pattern and a Western-style high-fat diet inducing pronounced phase delays and rhythm loss relative to low-fat diet controls. Both disruptions paralleled alterations in hepatocyte nuclear factor 4α (*Hnf4a*), newly implicating HNF4α as a potential mediator of ALDH2 circadian instability. In humans, ALDH2 and CYP2E1 exhibited conserved but phase-inverted circadian rhythms across multiple tissues relative to mice, and, importantly, night-shift workers showed markedly dampened and phase-shifted ALDH2 rhythms in peripheral blood mononuclear cells, providing the molecular link between occupational circadian misalignment and impaired acetaldehyde detoxification. Collectively, our detailed and innovative analytical approach reveals gene- and tissue-specific circadian regulation of alcohol-metabolizing enzymes, identifies ALDH2 as uniquely vulnerable to circadian misalignment, underscores the importance of circadian timing for optimal hepatic detoxification and resistance to tissue injury, and suggests that monitoring circadian rhythms could help tailor individualized advice on alcohol consumption for shift workers and populations with irregular sleep schedules, informing precision medicine approaches for alcohol-related disorders.

## 1. Introduction

The circadian clock is a fundamental biological timing system that generates near-24 h rhythms in physiological, biological and behavioral processes, such as sleep–wake cycles, hormone secretion, cellular repair and metabolism [1,2]. In mammals, the central pacemaker for the circadian clock resides in the brain in the suprachiasmatic nucleus (SCN). To regulate timing, neurons and astrocytes of the SCN possess cell-autonomous molecular clocks regulated by transcription-translation feedback loops [3]. More specifically, the BMAL1-CLOCK transcriptional activator complex induces rhythmic expression of its repressors, period 1/2/3 (PER1/PER2/PER3) and cryptochrome 1/2 (CRY1/CRY2), while a secondary loop involving the nuclear receptors REV-ERBα/β (also known as NR1D1/NR1D2) and RAR-related orphan receptor α/β (RORα/RORβ) rhythmically represses or activates *Bmal1* transcription to stabilize and reinforce circadian oscillations [4]. Using these feedback loops, along with neural, hormonal, and environmental/behavioral cues, the central pacemaker coordinates the peripheral clocks present in nearly all organs and cell types and aligns internal physiology with the external/environmental factors like the light–dark cycle [5,6]. These delicate processes are the critical steps for seamless interaction of the host with the environment that results in preparing the host to adjust to the environmental/behavioral changes. It is thus not surprising that disruption of the circadian homeostasis should impact the host resiliency and increase the vulnerability of the host that could lead to pathologies.

Large-scale genomic studies show that nearly half of all mammalian genes exhibit circadian rhythmicity in at least one tissue, with the specific rhythmic gene sets differing markedly across tissues and species [7,8,9]. In the liver, the molecular clock governs roughly 10–15% of the transcriptome, orchestrating daily rhythms in xenobiotic metabolism, energy balance, and detoxification [10,11]. Recent multi-omics studies further show that rhythmic changes in the levels of proteins and metabolites, as well as transcripts, are readily reprogrammed by circadian disruptions, such as sleep loss [12,13], a high-fat diet [14,15,16], night-shift work [17,18,19,20] and more relevant to this study, alcohol consumption [21,22].

Ethanol (alcohol) is principally metabolized in the liver via a two-step enzymatic pathway. It is first oxidized to acetaldehyde, primarily by alcohol dehydrogenase (ADH) along with contributions from cytochrome P450 2E1 (CYP2E1) [23]. Acetaldehyde, a highly reactive and toxic intermediate, is subsequently converted to acetate by aldehyde dehydrogenase (ALDH), predominantly ALDH2 with a minor contribution from ALDH1B1, within the mitochondria [24]. Notably, individuals carrying the inactive ALDH2*2 mutant allele experience acetaldehyde accumulation, leading to the alcohol flush reaction and elevated risk of alcohol-related diseases and cancer [25,26,27,28]. Moreover, we recently demonstrated that ALDH2 protects against binge alcohol-induced gut leakiness, endotoxemia, and acute liver injury via the gut-liver axis, as well as subsequent gut and brain injury, underscoring its central role in aldehyde detoxification and systemic protection [29,30]. In contrast, CYP2E1 contributes modestly to alcohol metabolism (alongside ADH and others) during modest amounts of alcohol consumption. In contrast, under conditions of chronic alcohol intake, CYP2E1 is strongly induced, and its catalytic activity leads to significant generation of reactive oxygen species (ROS) that contribute to oxidative stress, lipid peroxidation, post-translational protein modifications, and tissue injury (especially in the liver and gut) [31,32,33,34]. These findings emphasize the importance of balancing protective ALDH2-driven acetaldehyde clearance with the potentially harmful, CYP2E1-driven oxidative burden during chronic alcohol consumption.

Notably, previous studies have suggested that circadian rhythms and alcohol metabolism are tightly interconnected and contribute to alcohol-associated organ damage [35,36,37,38]. Indeed, an earlier animal study showed that alcohol clearance and toxicity varied depending on the time of day at which the alcohol was consumed [39]. Additional studies have also shown that alcohol exposure itself alters circadian clock function. For example, ethanol induces intestinal CYP2E1-dependent oxidative stress that upregulates the expression of CLOCK and PER2 proteins, thereby increasing gut permeability and liver injury [31,40]. Chronic alcohol consumption also markedly disrupts liver clock gene rhythms while leaving the SCN largely unaffected, indicating tissue-specific vulnerability of circadian regulation to alcohol [41]. On the other hand, circadian disruption, whether genetic (*Clock* Δ19) or environmental (repeated phase shifts, modeling shift work), not only worsens alcohol-induced gut and liver injury but also drives tissue-specific transcriptional changes that heighten susceptibility to alcohol-associated pathology and increase alcohol drinking [42,43,44]. Moreover, recent studies have shown that alcohol intake, especially when combined with circadian misalignment from light–dark shifts or wrong-time eating, disrupts central-peripheral clock coordination and weakens intestinal barrier resilience through microbiota-mediated mechanisms, thereby amplifying alcohol-related tissue injury [45,46,47]. Despite these advances in understanding the physiological and pathological links between circadian rhythms and alcohol consumption, the molecular mechanisms behind circadian regulation of the ADH-CYP2E1-ALDH2 pathway (the major oxidative alcohol metabolic pathway through which ethanol is converted to acetate), the dependence of this pathway on core clock mechanisms versus systemic cues, and the susceptibilities of individual metabolic enzymes to circadian disruptions have yet to be clearly defined.

Therefore, in the present study, we systematically mapped the circadian regulation of the ADH–CYP2E1–ALDH2 pathway in the liver, determined which components are governed by the core molecular clock versus external timing cues, and tested the sensitivity of each enzyme’s rhythm to circadian disruption. In addition, we conducted an integrative, cross-species analysis of publicly available circadian transcriptome datasets, spanning from genetic (clock gene knockout) and environmental perturbations (e.g., sleep deprivation or western high-fat diet) in mice to simulate real-world circadian misalignment (night-shift work) in humans, focusing on ADH1, CYP2E1, ALDH2, and ALDH1B1. We found that these enzymes exhibited distinct, gene-specific rhythmic profiles, with *ALDH2* and *CYP2E1* showing conserved, robust diurnal oscillations whose phases were opposite in mouse and human tissues, and *ADH1* and *ALDH1B1* showing inconsistent or non-rhythmic expression patterns, particularly in liver tissue. Notably, *Aldh2* and *Cyp2e1* rhythms in liver tissue persisted in global *Bmal1*-deficient and *Clock* mutant mice under light–dark conditions but required BMAL1 for rhythmicity in constant darkness (free run, intrinsic circadian rhythms), revealing both light- and clock-dependent layers of regulation. Among these enzymes, ALDH2 emerged as the most sensitive to environmental circadian disruption, showing pronounced dysregulation with sleep deprivation, a high-fat diet, and human shift-work schedules. Together, these findings provide an integrated view of circadian control across the ADH-CYP2E1-ALDH2 pathway and highlight the molecular mechanisms of daily and disruption-induced fluctuations in alcohol detoxification capacity and the molecular underpinning of increased susceptibility to alcohol associated pathologies in circadian disrupted rodents and humans.

## 2. Results

### 2.1. Alcohol-Metabolizing Enzymes Exhibit Diurnal Rhythms in Mouse Liver, with Aldh2 and Cyp2e1 Showing Robust Oscillations

**Figure 1:**
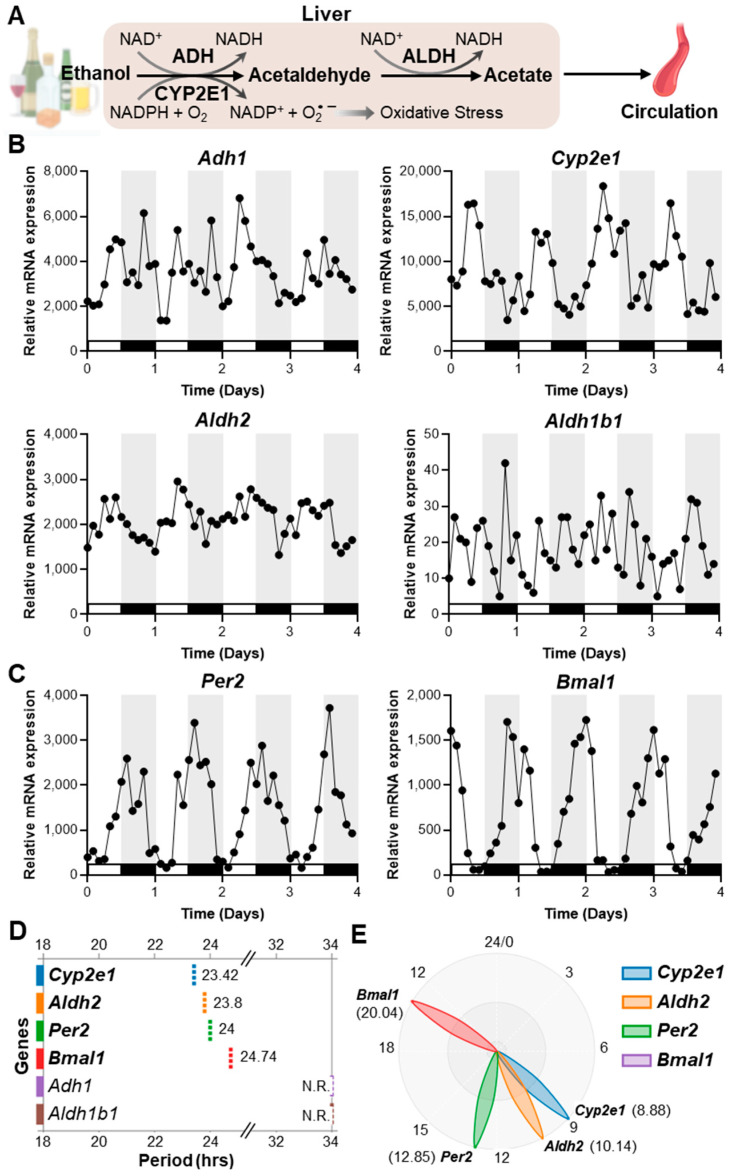
Alcohol-metabolizing enzymes exhibit diurnal oscillations in mouse liver. (**A**) Schematic illustration of hepatic alcohol metabolism. In the liver, alcohol is first converted to acetaldehyde, primarily through oxidative reactions catalyzed by alcohol dehydrogenase (ADH) and cytochrome P450-2E1 (CYP2E1). Acetaldehyde is subsequently oxidized by mitochondrial aldehyde dehydrogenase 2 (ALDH2) and its isoforms to form acetate. ADH and ALDH2 enzymes use nicotinamide adenine dinucleotide (NAD^+^) as an electron acceptor, generating NADH, whereas the CYP2E1 pathway consumes nicotinamide adenine dinucleotide phosphate (NADPH) and molecular oxygen (O_2_) to produce NADP^+^ and reactive oxygen species (O_2_^•−^), thereby contributing to oxidative stress. (**B**,**C**) Temporal mRNA expression profiles of the indicated alcohol-metabolizing enzymes ((**B**): *Adh1*, *Cyp2e1*, *Aldh2*, *Aldh1b1*) and circadian clock genes ((**C**): *Per2*, *Bmal1*) in the livers of male mice (10–14 weeks of age), collected every 2 h over a 94 h period under 12 h light/12 h dark conditions with ad libitum feeding. Data were generated from publicly available global RNA-seq datasets (GSE73554; [48]). (**D**,**E**) Period (**D**) and phase (**E**) estimates of mRNA rhythmicity corresponding to the profiles in panels (**B**,**C**). Analyses were performed using the BioDare2 rhythm analysis platform (https://biodare2.ed.ac.uk/; accessed to 20 November 2025). Genes for which no significant rhythmicity was detected are labeled as N.R. (non-rhythmic).

To investigate the baseline circadian expression patterns of oxidative alcohol-metabolizing enzymes, we re-analyzed a global RNA-seq dataset from mouse livers collected every 2 h over 94 h under standard 12 h light/12 h dark (LD) conditions (lights on at ZT0 and off at ZT12) with ad libitum feeding [48]. Rhythm analysis using BioDare2 revealed notable differences among enzymes in the major oxidative alcohol metabolism pathway (Figure 1).

Under the alcohol-naïve conditions, *Cyp2e1* displayed the most robust circadian oscillations, with a period of 23.42 h and amplitude variations of 2–3 fold between the peaks and troughs of expression (Figure 1B,D). *Aldh2* exhibited similarly strong rhythmicity, with a period of 23.8 h (Figure 1B,D). The levels of both enzyme transcripts peaked during the light phase (when mice are in their inactive/rest phase) and reached their lowest levels during the dark (active) phase (Figure 1B). The oscillation phases of *Aldh2* and *Cyp2e1* were also closely aligned with the rhythm of the canonical clock gene *Per2* and approximately anti-phasic to the *Bmal1* rhythm, suggesting that these enzyme cycles are integrated with the core clockwork (Figure 1B,E). In contrast, *Adh1* displayed inconsistent daily rhythmic patterns and did not meet the strict statistical criteria for significant rhythmicity. Furthermore, *Aldh1b1* was essentially arrhythmic, showing no discernible 24 h oscillation in our time-course analysis (Figure 1B,D). These observations were consistent with additional multi-tissue cycling analyses from the Circadian Expression Profiles Database (CircaDB) [7,49] (Figure S1). In this analysis, *Aldh2* was more robustly rhythmic in the liver compared with *Adh1* or *Cyp2e1*, whereas *Aldh1b1* was non-rhythmic across all tissues examined (Figure S1). Notably, *Adh1* exhibited pronounced rhythmicity in heart, brown adipose, and white adipose tissues, while *Cyp2e1* showed stronger oscillations in kidney, aorta, and adrenal gland (Figure S1), implicating tissue-specific circadian regulation of redox, aldehyde, and xenobiotic stress–handling pathways beyond classical hepatic alcohol metabolism. These results indicate that circadian regulation of the alcohol metabolic pathway is highly gene- and tissue-specific, with *Aldh2* and *Cyp2e1* displaying dominant rhythmic regulation that likely shapes daily variation in hepatic alcohol/aldehyde detoxification capacity.

### 2.2. Light–Dark Cycles Preserve Aldh2 and Cyp2e1 Rhythmicity, Even in the Absence of BMAL1/CLOCK-Mediated Core Clock Regulation

**Figure 2:**
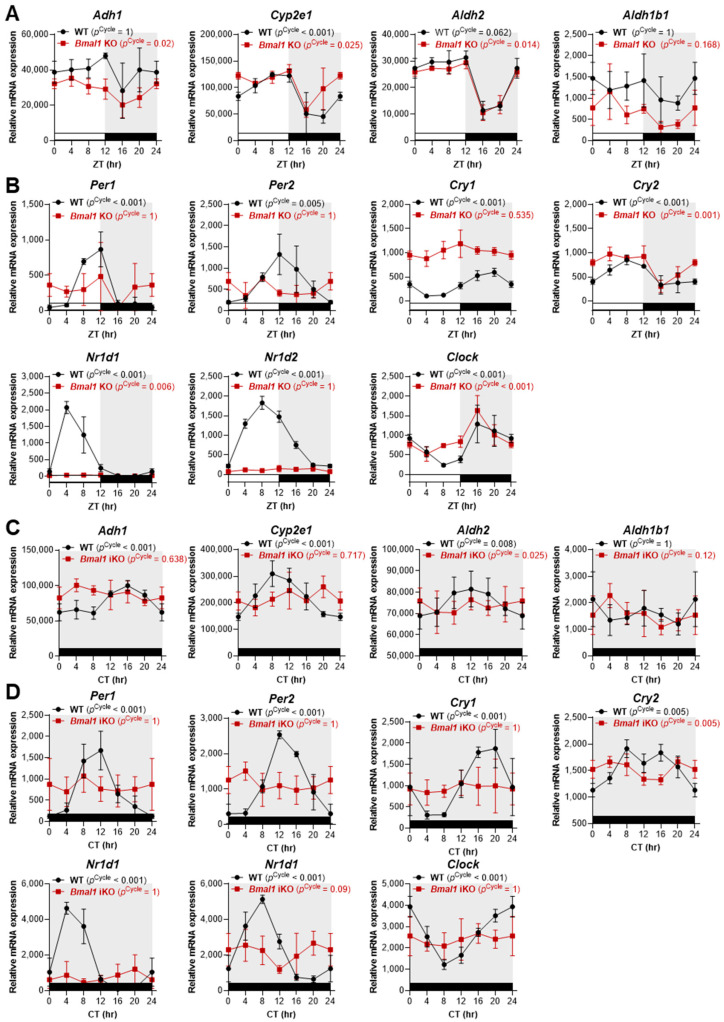
Light–dark cycles preserve *Aldh2* and *Cyp2e1* rhythmicity in *Bmal1*-deficient mice. (**A**,**B**) Time-series mRNA expression profiles of the indicated alcohol-metabolizing enzymes ((**A**): *Adh1*, *Cyp2e1*, *Aldh2*, *Aldh1b1*) and core circadian clock genes ((**B**): *Per1*, *Per2*, *Cry1*, *Cry2*, *Nr1d1*, *Nr1d2*, *Clock*) in livers from wild-type (WT; *n* = 3 per time point) and conventional *Bmal1* knockout (KO; *n* = 3 per time point) mice housed under 12 h light/12 h dark conditions (lights on at ZT0 and off at ZT12) with ad libitum feeding. Samples were collected every 4 h. (**C**,**D**) Time-series mRNA expression profiles of the indicated alcohol-metabolizing enzymes (**C**) and core clock genes (**D**) in livers from WT (*n* = 4 per time point) and postnatal tamoxifen-induced *Bmal1* knockout (iKO; *n* = 4 per time point) mice housed under constant darkness. Samples were collected every 4 h (circadian time [CT]). MetaCycle/JTK_Cycle rhythm analysis results for each target gene and genotype are indicated within the graphs. A MetaCycle/JTK_Cycle *p* < 0.05 denotes significant 24 h rhythmicity. Data were generated from publicly available global RNA-seq datasets (GSE70499; [50]).

To determine whether the circadian rhythmicity of oxidative alcohol-metabolizing enzymes requires the core clock transcription factor BMAL1, we analyzed RNA-seq data from wild-type (WT) and *Bmal1* knockout (KO) mouse livers under two distinct conditions: 12 h light–dark (LD) (using conventional *Bmal1* KO mice) and constant darkness (DD; using postnatal tamoxifen-induced *Bmal1* KO mice) [50] (Figure 2).

Under 12 h LD conditions, wild-type (WT) mice displayed strong circadian oscillations for *Cyp2e1* (MetaCycle/JTK_Cycle *p* [*p*^Cycle^] < 0.001) and moderate oscillations for *Aldh2* (*p*^Cycle^ = 0.062). Remarkably, in conventional *Bmal1* KO mice, both *Cyp2e1* and *Aldh2* maintained their rhythmic expression patterns despite the complete absence of Bmal1 (Figure 2A). This rhythmicity persisted even though many core clock genes (*Per1*, *Per2*, *Cry1*, *Cry2*, *Nr1d1*, *Nr1d2*) lost their rhythmicity in the KO animals, with the exception of *Clock*, which maintained its expression pattern (Figure 2A,B). *Adh1* showed weak rhythmicity that was Bmal1-independent, while *Aldh1b1* remained arrhythmic in both WT and KO mice (Figure 2A). Consistent with these results, additional analysis of the *Clock* Δ19 mutant (*Clock* MT) dataset from CircaDB revealed that *Aldh2* and *Cyp2e1* retained diurnal expression patterns (*p*^Cycle^ < 0.05) in *Clock* MT mice that were similar to what was observed in WT liver under LD conditions (Figure S2A), while *Per2* and *Bmal1* rhythms were markedly disrupted in *Clock* MT mice (Figure S2B). In contrast to the 12 h LD conditions, markedly different expression patterns of alcohol-metabolizing enzymes emerged when the mice were housed in DD conditions. In WT mice that were maintained in the dark, *Adh1*, *Cyp2e1*, and *Aldh2* continued to exhibit significant daily oscillations (*p*^Cycle^ < 0.001 for *Adh1* and *Cyp2e1*, *p*^Cycle^ = 0.008 for *Aldh2*), while *Aldh1b1* remained arrhythmic (Figure 2C). However, these rhythms were completely abolished in postnatal, tamoxifen-induced *Bmal1* KO mice, paralleling the loss of rhythmicity of core clock gene expression (Figure 2C,D). These results suggest that systemic cues (likely originating from the SCN via neural and hormonal signals) can impose rhythmic expression on these metabolic genes when external zeitgebers (light–dark cycles) are present, effectively ‘masking’ the loss of intrinsic clock-driven rhythmicity.

### 2.3. Acute Sleep Deprivation Differentially Disrupts Alcohol-Metabolizing Enzyme Rhythms in Mouse Liver

**Figure 3:**
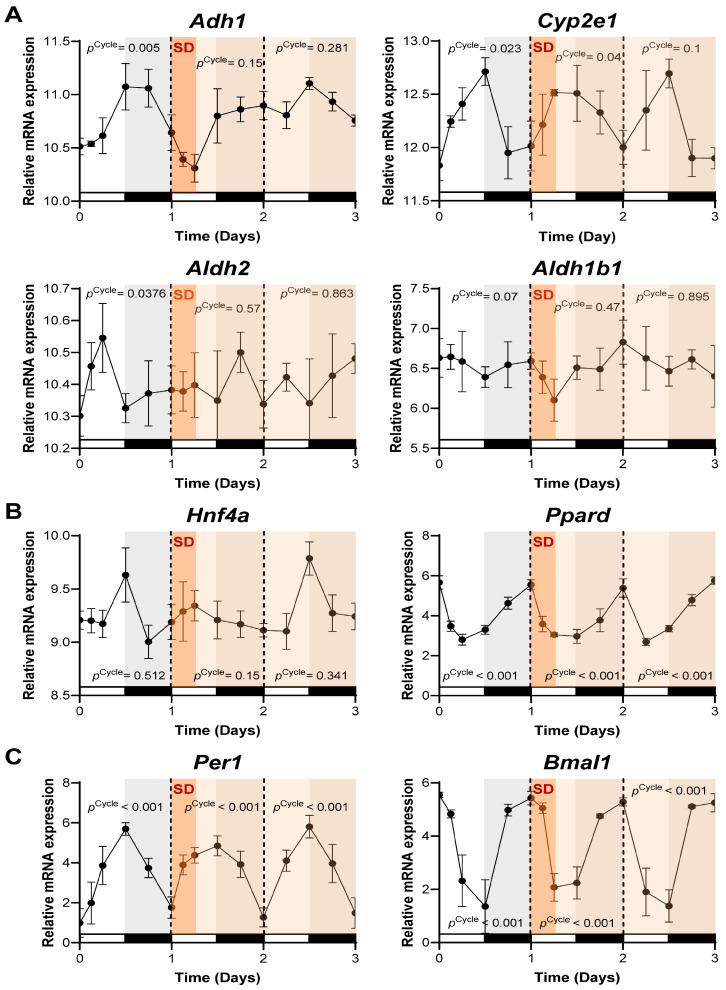
Acute sleep deprivation differentially alters the diurnal oscillations of alcohol-metabolizing enzymes in mouse liver. (**A**–**C**) Time-series mRNA expression profiles of alcohol-metabolizing enzymes ((**A**): *Adh1*, *Cyp2e1*, *Aldh2*, *Aldh1b1*), Aldh2-regulating genes ((**B**): *Hnf4a*, *Ppard*), and core circadian clock genes ((**C**): *Per1*, *Bmal1*) in the livers of 10–12-week-old male mice before (Day 0–1), during (on Day 1), and after (Days 1–3, light orange) acute sleep deprivation (SD; dark orange). Mice were sleep-deprived for 6 h from ZT0 to ZT6 on Day 1 during the light (resting) phase. Mice were housed under 12 h light/12 h dark conditions (lights on at ZT0 and off at ZT12) with ad libitum feeding. Liver samples were collected every 4 h. Each time point included 3–4 biological replicates, and eight ZT0 controls were collected from two animal batches. MetaCycle/JTK_Cycle rhythm analysis results (*p*^Cycle^) for each gene and day are shown within the graphs. *p*^Cycle^ < 0.05 indicates significant 24 h rhythmicity. Data were generated from publicly available global RNA-seq datasets (GSE262410; [13]).

Having established the circadian expression patterns of alcohol-metabolizing enzymes under normal and genetically perturbed conditions, we next examined how acute environmental disruption affects these rhythms by analyzing existing RNA-seq data. For these studies, mice were housed under standard 12 h LD conditions, and their livers were collected for RNA-seq analysis before (Day 0–1), during (Day 1–2), and after (Day 2–3) a 6 h acute sleep deprivation (SD) period (ZT0–ZT6 on Day 1) [13] (Figure 3).

As shown in the rhythm analysis in Figure 3A, *Adh1* (*p*^Cycle^ = 0.005), *Cyp2e1* (*p*^Cycle^ = 0.023), and *Aldh2* (*p*^Cycle^ = 0.037) exhibited significant rhythmicity prior to SD (Day 0–1), whereas *Aldh1b1* ((*p*^Cycle^ = 0.486) did not reach statistical significance. Following acute SD (Day 1–2), *Adh1* (*p*^Cycle^ = 0.150) and *Aldh2* (*p*^Cycle^ = 0.57) immediately lost rhythmicity, while *Cyp2e1* maintained significant oscillations (*p*^Cycle^ = 0.04), demonstrating differential vulnerability of the expressions of these enzymes to sleep loss. Notably, *Aldh2* displayed the most disrupted post-SD profile, with an immediate dampening of expression and a highly unstable, arrhythmic pattern (*p*^Cycle^ = 0.863) across Days 1–3. In contrast, *Adh1* and *Cyp2e1* showed partial recovery of their pre-SD rhythmic patterns by Day 2–3, although this restoration was not statistically significant and differed in magnitude between the two genes (*p*^Cycle^ = 0.1 ~ 0.28).

To understand the molecular basis for *Aldh2* vulnerability to circadian disruption, we examined the rhythmic profiles of its known transcriptional regulators in parallel. *Hnf4a*, a liver-specific transcriptional regulator of *Aldh2* [51], showed dampened expression immediately after SD, followed by recovery of its rhythmicity to pre-SD levels at Day 2–3. In contrast, *Ppard*, another identified activator of *Aldh2* transcription [52,53], retained robust oscillations throughout the experimental period, with strong statistical support for rhythmicity (*p*^Cycle^ < 0.001 for all days) (Figure 3B). This temporal correlation between *Hnf4a* disruption and *Aldh2* instability suggests that HNF4α plays a more significant role than *Ppard*, in SD-induced alterations in *Aldh2* rhythmicity. Interestingly, most core clock genes (*Per1*, *Per2*, *Bmal1*, *Cry1*, *Cry2*, and *Nr1d2*) maintained stable rhythmicity following SD, with the notable exceptions of *Clock* and *Nr1d1*, which exhibited disrupted or markedly dampened oscillations (Figure 3C and Figure S3). Collectively, these data indicate that the temporal regulation of alcohol-metabolizing enzymes, particularly *Aldh2*, is highly sensitive and vulnerable to the effects of sleep loss.

### 2.4. A High-Fat Diet Induces Rhythm Alterations in Aldh2 Expression via Hnf4a-Linked Phase Delays in Mouse Liver

**Figure 4:**
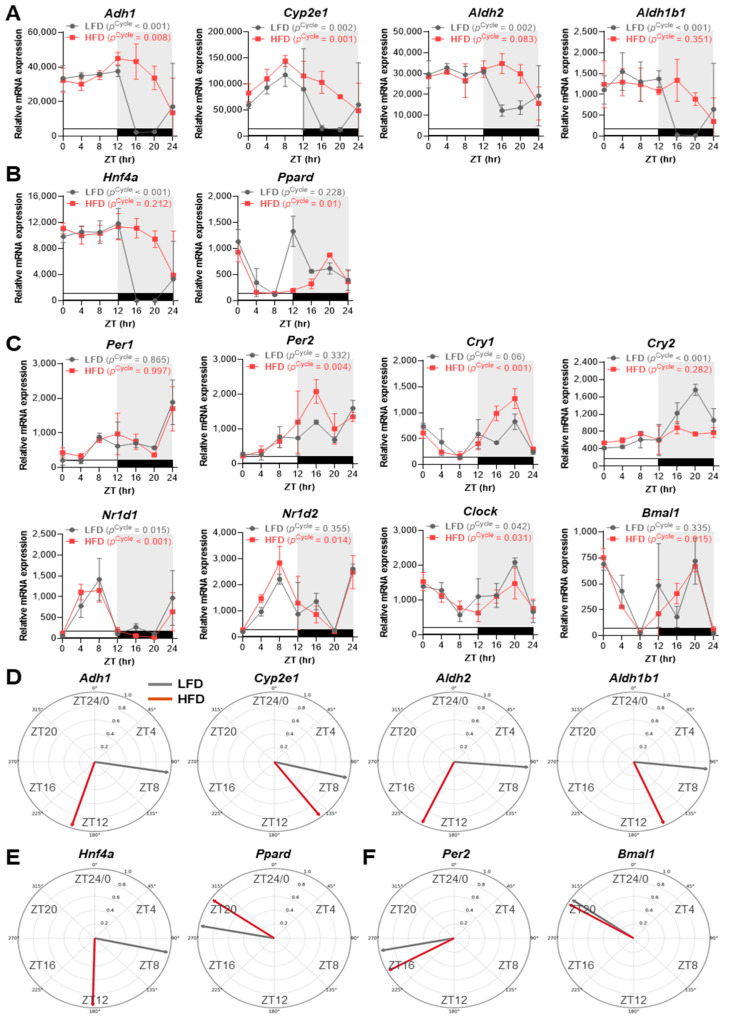
Feeding a high-fat diet (HFD) reprograms the circadian expression and phase timing of hepatic alcohol-metabolizing enzymes in mouse liver. (**A**–**C**) Temporal mRNA expression profiles for alcohol-metabolizing enzymes (*Adh1*, *Cyp2e1*, *Aldh2*, *Aldh1b1*), transcriptional regulators (*Hnf4a*, *Ppard*), and core clock genes (*Per2*, *Bmal1*) in the livers of 6-week-old female mice after three weeks on either a standard low-fat diet (LFD; gray) or a high-fat diet (HFD; red), initiated at 3 weeks of age prior to tissue collection (*n* = 3 per ZT for each diet). Mice were housed under a 12 h light/12 h dark cycle with lights on at ZT0 and off at ZT12, fed ad libitum, and sampled every 4 h over a 24 h period. Data represent means ± SEM. White bars denote the light phase, and black bars denote the dark phase. JTK_Cycle *p*-values (*p*^Cycle^) for rhythmicity under LFD and HFD are shown above each panel. (**D**–**F**) Phase-vector diagrams summarizing the circadian peak phases for the indicated alcohol-metabolizing enzymes (**D**), regulatory transcription factors (**E**), and core clock components (**F**) under LFD (gray) and HFD (dark red) conditions were derived from the MetaCycle/JTK_Cycle analyses. Each arrow denotes the acrophase (circadian peak) of maximal expression (ZT0 is positioned at 12 o’clock; clockwise progression). Data were generated from publicly available global RNA-seq datasets (GSE218932; [16]).

To investigate how chronic metabolic disruption influences the circadian regulation of alcohol metabolism, we analyzed liver RNA-seq data from female mice placed on a high-fat diet (HFD) at 3 weeks of age for 3 weeks (tissue collected at ~6 weeks), with samples collected every 4 h over 24 h under a 12 h LD cycle and compared with age-matched low-fat diet controls (LFD) [16]. Under LFD feeding conditions, *Adh1*, *Cyp2e1*, *Aldh2*, and *Aldh1b1* all exhibited robust diurnal fluctuations (*p*^Cycle^ < 0.01) characterized by high expression during the light (resting) phase and low expression during the dark (active) phase (Figure 4A).

Notably, this pattern likely reflects strong zeitgeber-driven oscillations in these very young mice, whose endogenous circadian clocks remain more flexible to light–dark cues compared with the more established clocks of typical 8–10-week-old mice used in other studies [54]. HFD exposure differentially altered these oscillations. Indeed, *Adh1* and *Cyp2e1* maintained significant rhythmicity (*p*^Cycle^ < 0.01), while *Aldh2* and *Aldh1b1* lost statistical rhythmicity (*p*^Cycle^ > 0.05) and showed pronounced phase-delayed shifts in peak expression (Figure 4A). Similar HFD-induced phase delays were also observed for *Hnf4a*, in parallel with the rhythmic disruption of *Aldh2*, but not for *Ppard* (Figure 4B), reflecting the regulatory patterns also seen under sleep-deprived conditions (Figure 3A,B). Our analyses further showed that core clock genes displayed mixed responses to HFD, with several rhythms being strengthened (*Per2*, *Cry1*, *Nr1d2*, *Clock*, *Bmal1*), some rhythms being minimally affected (*Per1*, *Nr1d1*), and *Cry2* rhythmicity being markedly dampened and abolished (Figure 4C). Phase-vector analysis further confirmed these results, revealing that, compared to *Adh1*, *Cyp2e1*, and *Aldh1b1*, *Aldh2* exhibited the largest phase-delay shift, which was closely aligned with *Hnf4a*, while *Per2* and *Bmal1* showed minimal phase-shift responses under HFD feeding conditions (Figure 4D–F). Consistent with these results, analysis of an independent liver RNA-seq dataset from male mice that initiated HFD feeding at 6 weeks of age and were maintained on HFD for 10 weeks [14] similarly revealed that *Aldh2* exhibited the largest HFD-induced alteration in diurnal expression, comparable only to *Hnf4a*, and exceeding the changes observed for *Adh1*, *Cyp2e1*, and *Ppard* (Figure S4). These findings show that, similar to SD, HFD differentially alters the rhythms of alcohol-metabolizing enzymes. In particular, the hepatic circadian regulation of *Aldh2* was found to be most profoundly affected, likely due to altered *Hnf4a* dynamics.

### 2.5. ALDH2 and CYP2E1 Display Conserved Circadian Rhythmicity Across Human Tissues

**Figure 5:**
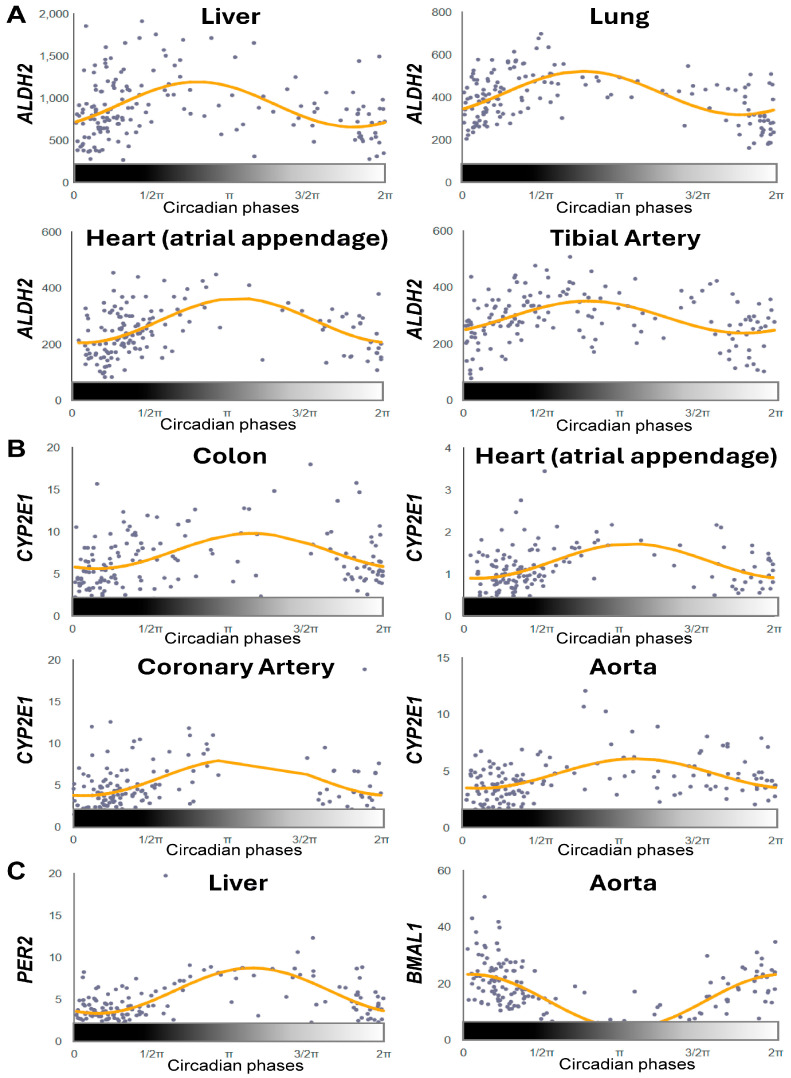
*ALDH2* and *CYP2E1* display diurnal oscillations across multiple human tissues. (**A**–**C**) Rhythmic diurnal expression profiles of *ALDH2* (**A**), *CYP2E1* (**B**), and the core clock genes *PER2* and *BMAL1* (**C**) in the indicated human tissues. Each dot represents normalized gene expression from an individual sample plotted against its inferred circadian phase. The orange line represents the fitted harmonic regression curve modeling rhythmic expression across the 24-h cycle. Circadian phases are shown using a 24 h circular scale: phase 0 (or 2π) marks the start of the biological night (i.e., 9–10 PM), π/2 corresponds to midnight during sleep (i.e., 3–4 AM), π indicates the start of the biological day (i.e., 9–10 AM), and 3π/2 represents midday during the active wake period (i.e., 3–4 PM). Data were obtained from the Circadian Expression Profiles Database (CircaDB; http://circadb.hogeneschlab.org/human, 1 October 2025) and analyzed using an FDR-adjusted *p*-value cutoff of 0.05 and a relative amplitude (rAMP) > 0.1.

To evaluate the translational relevance of our mouse findings, we examined the circadian expression patterns of alcohol metabolism enzymes in human tissues using CircaDB [9,49]. Human tissue-specific circadian profiling (false discovery rate [FDR] < 0.05; relative amplitude rAMP > 0.1) revealed that *ALDH2* and *CYP2E1*, but not *ADH1* or *ALDH1B1*, exhibited clear diurnal rhythmicity across multiple tissues (Figure 5A,B).

*ALDH2* showed robust oscillations in the liver, lung, heart (atrial appendage), and tibial artery (Figure 5A). *CYP2E1* displayed significant rhythms in colon, coronary artery, aorta, and heart atrial tissue (Figure 5B). In addition, *ALDH2* and *CYP2E1* shared similar rhythmic phases, with expression patterns aligned to *PER2* and anti-phasic to *BMAL1* in humans, mirroring the mouse liver findings. Importantly, we observed an inverse phase relationship between the nocturnal (mouse) and diurnal (human) species. In mouse liver, *Aldh2* and *Cyp2e1* showed peak expression during the light (resting) phase and lower expression during the dark (active) phase (Figure 1, Figure 2, Figure 3 and Figure 4). In human tissues, particularly human liver, *ALDH2* expression peaked during the dark (inactive) phase (biological night) and decreased during the light (active) phase (biological day) (Figure 5A). This 12 h phase shift is consistent with the behavioral inversion between nocturnal and diurnal species while maintaining the relationship with rest/activity cycles. This tissue survey aligns with the prior mouse results and suggests that robust circadian regulation of alcohol-metabolizing enzymes is conserved specifically for *ALDH2* and *CYP2E1*.

### 2.6. Night-Shift Work Markedly Disrupts ALDH2 Rhythmicity in Human PBMCs

**Figure 6:**
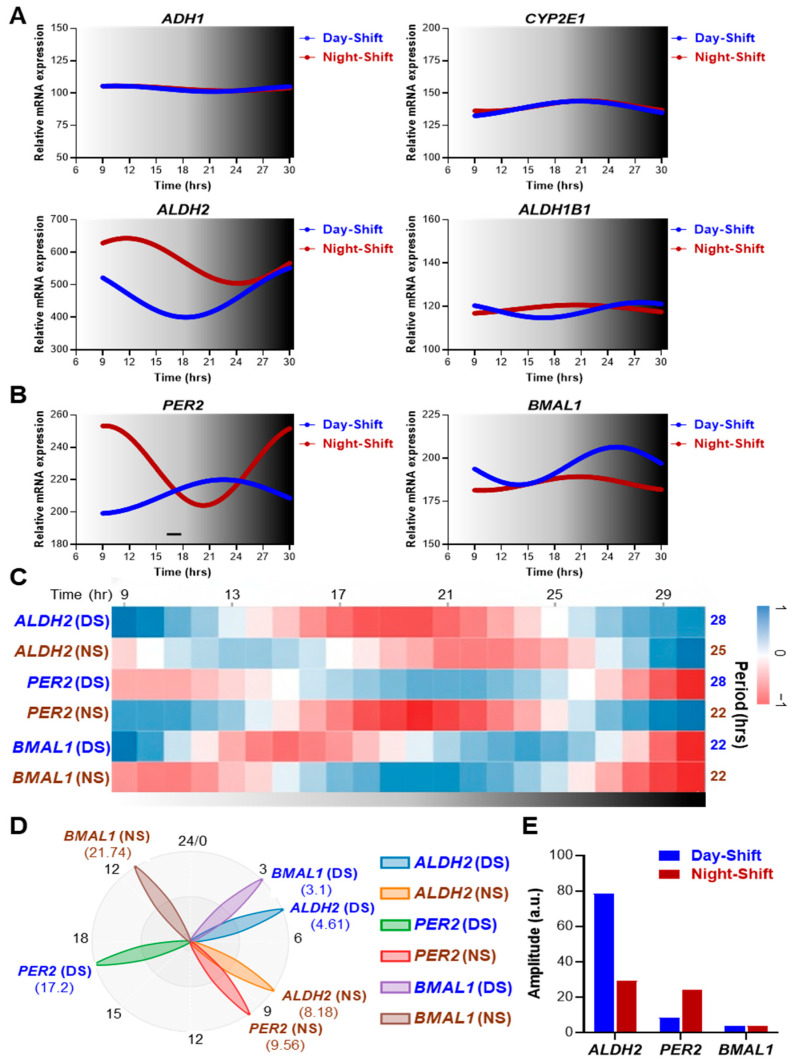
Night-shift work strongly alters daily *ALDH2* gene expression in human PBMCs. (**A**,**B**) Temporal mRNA expression profiles of alcohol-metabolizing enzymes ((**A**): *ADH1*, *CYP2E1*, *ALDH2*, *ALDH1B1*) and core circadian clock genes ((**B**): *PER2*, *BMAL1*) in peripheral blood mononuclear cells (PBMCs) isolated every three hours from night-shift (NS, dark brown) and day-shift (DS, blue) nurses over a 24 h period on a day off under controlled laboratory conditions (isocaloric, time-matched meals, and dim red light). (**C**) Heatmap visualization of the rhythmic changes in *ALDH2*, *PER2*, and *BMAL1* expression over 24 h in DS and NS PBMCs, including estimated period values derived from the time-course data in (**A**,**B**). (**D**,**E**) Phase (**D**) and amplitude (**E**) estimates of transcript rhythmicity corresponding to the heatmap profiles in (**C**). Rhythm analyses for panels (**C**–**E**) were performed using the BioDare2 platform (https://biodare2.ed.ac.uk/; accessed to 20 November 2025). Data were generated using a publicly available global RNA-seq dataset (GSE122542; [18]).

To assess how chronic circadian disruption affects alcohol-metabolizing enzyme expression in humans, we analyzed circadian transcriptome data from peripheral blood mononuclear cells (PBMCs) of day-shift and night-shift hospital nurses [18] (Figure 6).

PBMCs were collected every three hours over 24 h on a day off under controlled laboratory conditions (isocaloric, time-matched meals, dim red light). Among the alcohol-metabolizing enzymes examined, *ALDH2*, but not *ADH1*, *CYP2E1*, or *ALDH1B1*, displayed a clear and detectable rhythmic pattern, reflecting the tissue-specific nature of the expression profiles of these enzymes (Figure 6A). In day-shift subjects, *ALDH2* (period: 28 h) exhibited robust diurnal rhythmicity that was comparable to the rhythms of the core clock genes *PER2* (period: 28 h) and *BMAL1* (period: 22 h), with higher expression during the dark (inactive) phase, which is consistent with the human tissue patterns observed in the CircaDB data (Figure 6A–C). Notably, phase analysis showed that *ALDH2* reached its acrophase at 4.61 h, whereas *PER2* and *BMAL1* peaked at 9.56 h and 21.74 h, respectively (Figure 6D), indicating phase relationships in PBMCs that differ from those in other human tissues. In contrast, night-shift subjects displayed markedly altered *ALDH2* rhythmicity. In these subjects, the period was shortened to approximately 25 h, the peak phase was shifted to 8.18 h, and the amplitude was substantially reduced, compared to day-shift workers (Figure 6A–D). In addition, *ALDH2* amplitude was notably higher in day-workers compared to night-shifters, while amplitude differences between the two groups were less pronounced for *PER2* and minimal for *BMAL1* (Figure 6E). This pattern of specific *ALDH2* disruption was distinct from what was observed for the core clock genes, where *PER2* showed period changes (22 vs. 28 h) but maintained relative amplitude, and *BMAL1* maintained both period (~22 h) and amplitude across groups (Figure 6C,E). Importantly, *ADH1*, *CYP2E1*, and *ALDH1B1* did not exhibit comparable rhythmic alterations between the day- and night-shift workers (Figure 6A). Together, these findings indicate that *ALDH2* is uniquely sensitive to circadian misalignment, suggesting its particular vulnerability across tissues and species.

## 3. Discussion

In the present study, we examined the circadian regulation of oxidative alcohol-metabolizing enzymes and the impact of circadian disruption across species and experimental conditions. We have found that these enzymes exhibit gene- and tissue-specific circadian oscillations under dual regulation by intrinsic clock mechanisms and zeitgeber cues, with *ALDH2* emerging as the most vulnerable to sleep loss, a high-fat diet, and shift work. These findings indicate that time-of-day detoxification of acetaldehyde and other toxic lipid aldehydes is especially sensitive to circadian misalignment, which might explain the increased vulnerability of rodents and humans with disrupted circadian rhythms to alcohol-associated organ damage.

### 3.1. Robust Diurnal Rhythms in Aldh2 and Cyp2e1 Expression Indicate Circadian Regulation of Alcohol Detoxification Across Species

In our analysis, *Cyp2e1* and *Aldh2* exhibited the most robust and consistent diurnal rhythmicity across the mouse liver transcriptome datasets examined. In contrast, *Adh1* showed weak or nonsignificant rhythms in some studies (Figure 1B and Figure 2A) but significant oscillations in others (Figure 3A and Figure 4A), and *Aldh1b1* was uniformly non-rhythmic. Despite minor study-to-study differences, the collective evidence indicates that *Adh1*, *Cyp2e1*, and *Aldh2* generally show higher expression during the resting phase (day) and lower expression during the active phase (night) in mice. Consistent with this pattern, an earlier report demonstrated circadian rhythms in mitochondrial ALDH isozyme activity, with peak activity during the light (resting) phase and reduced activity during the dark (active) phase in male mice, although this fluctuation was absent in females [55]. Likewise, we observed analogous rhythms for *ALDH2* and *CYP2E1* in human tissues but with phases opposite of those observed in mice (higher expression during the night/resting period; Figure 5 and Figure 6). Given that peak *ALDH2* and *CYP2E1* expression aligns with the inactive phase, a conserved feature across species, it is tempting to speculate that alcohol metabolism and acetaldehyde detoxification may be intrinsically more efficient when the organism is at rest. Supporting this notion, mouse blood alcohol concentration (BAC) peaks at ZT15 and reaches its nadir at ZT7 [39]. Furthermore, ethanol-induced lethality shows strong circadian dependence, with a 5 mg/g dose causing 80% mortality at ZT24 but no deaths at ZT12, and even higher doses (5.57–6.5 mg/g) producing more rapid and frequent death during the dark (active) phase than during the light (resting) phase [39]. In contrast to these nocturnal patterns, humans, being diurnal, likely exhibit the opposite time-of-day pattern of alcohol metabolism, with greater metabolic efficiency exhibited during the evening resting phase, when *ALDH2* and *CYP2E1* expressions are higher than during the morning active phase (Figure 5). Consistent with this possibility, controlled chronopharmacology studies have shown that identical alcohol doses produce higher BACs when alcohol is consumed in the morning compared to the evening [56,57]. Similarly, chronotoxicology studies in zebrafish have demonstrated greater mortality and behavioral impairment when alcohol exposure occurs during the active phase rather than the resting phase [58]. Together, these observations strongly suggest that the timing of alcohol intake can significantly influence its pharmacokinetics and toxic effects, likely reflecting circadian regulation of its metabolic enzymes.

### 3.2. BMAL1-Independent Diurnal Rhythmicity of Cyp2e1 and Aldh2 Reveals Dual Intrinsic and Systemic Circadian Control

Importantly, our circadian analyses of datasets from *Bmal1* KO and *Clock* mutant mice showed that *Aldh2* and *Cyp2e1* retain rhythmic expression under LD conditions, despite the complete loss of core clock gene oscillations in these clock-deficient animals (Figure 2A,B and Figure S2A,B). Further rhythm analysis revealed that under DD conditions, *Cyp2e1*, *Aldh2*, and even *Adh1* (which is arrhythmic under LD) display *bona fide* circadian clock-controlled rhythms, each with gene-specific peak phases (Figure 2C,D). These findings are reminiscent of classic work from the 1980s showing that ALDH activity rhythms disappear in DD in the absence of an intact clock [55]. Mechanistically, prior studies provide clues as to why *Cyp2e1* and *Aldh2* exhibit particularly robust rhythmicity. HNF1α binds to the *Cyp2e1* promoter in a time-dependent manner, interacting alternately with the CRY1 repressor or the p300 coactivator in mouse liver cells [59]. *Aldh2*, in turn, has been identified as a direct BMAL1/CLOCK transcriptional target in mouse brain tissues, as it contains E-box motifs in its promoter [60]. Such control by these factors likely contributes to the strong circadian regulation of *Aldh2* in the liver as well. In support of this, our mouse liver data show that *Aldh2* transcript rhythms align more closely with *Per1* and *Per2* oscillations than with *Adh1* or *Cyp2e1* under DD (Figure 2C). In addition, their loss of rhythms in DD but preservation in LD even without BMAL1 strongly suggests that systemic signals, likely mediated by SCN-driven neural and hormonal outputs, can mask the absence of intrinsic clock function and impose apparent rhythmicity on these metabolic genes in liver (Figure 2A,C). This is reminiscent of a prior study showing that systemic cues can drive oscillations in a subset of hepatic genes even when the local circadian clock is impaired [61]. Thus, hepatic oxidative alcohol-metabolizing genes may lie at the intersection of intrinsic and extrinsic regulation, illustrating how clock-controlled genes can still be governed by systemic zeitgebers, even in the absence of a functional molecular clock.

### 3.3. Enhanced Vulnerability of Aldh2 Circadian Rhythms to Sleep Loss and a High-Fat Diet Suggests a Regulatory Role for HNF4α

Our analysis of circadian disruption revealed that *Aldh2* is the most vulnerable alcohol-metabolizing enzyme under both SD and HFD conditions. A single bout of SD was sufficient to dampen and desynchronize *Aldh2* expression for nearly two full cycles in mice (Figure 3A). Although *Cyp2e1* and *Adh1* were also affected by SD and HFD, their rhythms showed faster and more partial recovery, underscoring the gene-specific sensitivity of *Aldh2* to circadian disruption. Moreover, the tight temporal correspondence between *Hnf4a* dampening and *Aldh2* instability suggests that HNF4α is a potential mediator of this SD-induced disruption (Figure 3A,B). In contrast, *Ppard* maintained robust rhythmicity, indicating that not all *Aldh2* regulators are equally impacted. In support of this notion, a recent study reported that HNF4α mRNA and protein levels are rapidly suppressed by pro-inflammatory signals such as IL-6, IL-1β, and lipopolysaccharide (LPS), all of which are known to rise during sleep loss [62,63,64,65] accompanied by increased intestinal barrier dysfunction [66,67,68], thereby shifting the liver away from homeostatic functions and toward acute-phase gene programs [69]. These findings raise the possibility that acute SD may transiently suppress *Hnf4a* expression (Figure 3B) through cytokine-mediated pro-inflammatory signaling, attenuating HNF4α’s direct impact on the *Aldh2* promoter as well as its indirect modulation of CLOCK-BMAL1-driven E-box activity, thereby destabilizing *Aldh2* rhythmicity. A similar mechanism may be induced by a HFD, since with such a diet, *Aldh2* loses its rhythmicity and exhibits a marked phase delay that parallels phase-delayed shifts in *Hnf4a* but not *Ppard* expression, a pattern not observed for *Adh1* or *Cyp2e1* (Figure 4A and Figure S4). Together, these results support a model in which HNF4α-linked pathways act as key regulatory nodes connecting environmental perturbations to dysregulation of circadian *Aldh2* expression in mouse liver, with potential implications for detoxification capacity against alcohol/aldehyde and many other toxic agents.

Notably, previous studies have shown that HNF4α regulates numerous hepatic metabolism genes, is rhythmically expressed in mouse liver, and can trans-repress CLOCK-BMAL1 activity through direct physical interaction [70,71]. HNF4α has also been shown to repress BMAL1 in hepatocellular carcinoma, where BMAL1 and HNF4α display mutually incompatible rhythmicity [72], a relationship mirrored by the anti-phasic patterns observed in our data (Figure 3B,C and Figure 4B–D). Given this bidirectional regulatory interplay with the core clock, SD-induced disruption of HNF4α rhythmicity may contribute to the modest (*Per1*, *Per2*, *Bmal1*, *Cry1*, *Cry2*, *Nr1d2*) or more pronounced (*Clock*, *Nr1d1*) dampening of clock gene rhythms shortly after SD (Figure 3C and Figure S3), as well as the differential clock gene-specific effects observed with a HFD (Figure 4C,F).

### 3.4. Systemic Circadian Misalignment Perturbs ALDH2 Rhythms in Night-Shift Workers and May Undermine the Predictability of Alcohol Chronotoxicity

ALDH2 is a protective, antioxidative enzyme against mitochondrial dysfunction, organ damage, cancer, and aging-related neurodegeneration [30,73,74,75] while CYP2E1 is involved in producing reactive oxygen species (ROS), leading to elevated oxidative stress, mitochondrial dysfunction, and organ damage [76,77,78,79]. Having Cys in its active site [80], ALDH2 catalytic activity is frequently suppressed under oxidative stress after alcohol exposure, HFD, or other toxic agents [81,82,83,84,85]. The ALDH2 activity is suppressed by many agents or oxidative stress conditions, which are at least partially caused by increased CYP2E1, which promotes various posttranslational protein modifications, ER stress, mitochondrial dysfunction, impaired autophagy, gut leakiness, and organ damage [86]. Thus, antioxidant ALDH2 and prooxidant CYP2E1 play opposing roles in mitochondrial dysfunction and organ damage [87,88]. In addition, night shift workers are known to have elevated oxidative stress markers, such as high levels of ROS, lipid peroxides, increased DNA damage with reduced DNA repair and antioxidants like melatonin [89,90,91,92]. Based on this information, it is expected that the marked disruption of *ALDH2* expression in night-shift workers (and under resting phase in rodents) significantly increases the vulnerability to organ damage or dampens pre-existing conditions, especially after alcohol drinking or exposure to other potentially toxic agents, including acetaminophen, which is known to cause acute liver injury [93,94]. The additive or synergistic potentiation of harms in night-shift workers could result from the fact that these individuals already have leaky gut [68,95] with elevated levels of endotoxin LPS, which promotes the cell death signaling pathways and organ damage [66].

While PBMCs are not the primary site of alcohol metabolism, gene expression profiles in PBMCs are likely to reflect the patterns in the liver and other tissues. The pronounced alterations in *ALDH2* rhythms compared to the changes observed in *ADH1*, *CYP2E1*, and *ALDH1B1* in PBMCs of night-shift workers suggest that there are systemic effects of circadian misalignment that specifically affect *ALDH2* (Figure 5). This is consistent with PBMC transcriptome patterns that reflect broader physiological circadian states [96]. Notably, night-shift workers have been shown to have abnormal liver enzymes [97] and increased alcohol consumption patterns [98,99], which may, in turn, disrupt sleep timing/quality and health [100], creating a vicious cycle for elevated addiction and pathogenesis. Consistent with this, Swanson, Keshavarzian, and colleagues showed that shift workers with circadian misalignment exhibit significantly greater vulnerability to alcohol-induced intestinal permeability and elevated endotoxin levels compared to day workers consuming identical alcohol doses [68], indicating that circadian disruption amplifies alcohol-related barrier dysfunction through the gut-liver axis. Our observation that ALDH2, the critical protective enzyme in clearing toxic acetaldehyde and other lipid aldehydes, shows pronounced alterations in its circadian rhythms in night-shift workers’ PBMCs (Figure 6A–E) provides a plausible mechanistic explanation for their enhanced vulnerability. If peripheral disruption of *ALDH2* expression in PBMCs reflects similar disruption in intestinal and/or hepatic tissue, night-shift workers may experience dysregulated acetaldehyde clearance, potentially creating temporal windows of increased acetaldehyde accumulation during alcohol consumption that may lead to worse alcohol-induced toxicity. Our findings further underscore that although the timing of alcohol intake can markedly influence its pharmacokinetics and toxicity through circadian regulation of enzymes, such as ALDH2, this chronotoxicological relationship may become unreliable in individuals with misaligned or stressed circadian systems. Disrupted metabolic rhythm and the substantial inter-individual variability driven by different lifestyle factors, warrant further investigation to better understand when circadian timing of oxidative alcohol metabolism may lose its predictive value.

Several limitations of this study should be acknowledged. First, our study relies on publicly available transcriptome datasets, which preclude direct measurements of time-of-day enzyme expression and activity levels. The relationship between mRNA and protein levels may vary due to post-transcriptional regulation and post-translational protein modifications, and enzyme activity could be modulated by multiple factors beyond gene expression. Second, we noted minor inconsistencies in rhythmic gene detection across studies, particularly for *Adh1*, which exhibited significant oscillations in some datasets (Figure 3A and Figure 4A) but not in others (Figure 1B and Figure 2A). Notably, one prior study reported significant rhythms in *Adh1* and *Cyp2e1* but not *Aldh2* under LD conditions [41]. Such variability likely reflects the well-documented challenges in reproducing rhythmic gene expression patterns in liver transcriptome data, which can be influenced by differences in sampling intervals, sequencing depth, analytical methods, and biological factors including age, lighting, diet, and microbiome composition [101,102]. Third, the human shift worker data utilized PBMCs rather than hepatic tissue, limiting direct correlations with liver enzyme patterns. Fourth, we analyzed distinct experimental paradigms (acute 6 h SD vs. chronic HFD feeding vs. occupational shift work) that differ in temporal dynamics and may engage partially overlapping but not identical mechanisms. Fifth, we did not directly assess time-of-day-dependent alcohol metabolism, alcohol or acetaldehyde accumulation, or alcohol-induced tissue damage following alcohol intake at different circadian phases (active vs. resting) in either mice or human subjects under normal or circadian-disruptive conditions. Such measurements would provide functional validation of our transcriptomic findings across both animal and human models. Future studies should incorporate these functional assays to address this gap.

Despite these limitations, our study provides several unique contributions to the fields of chronobiology, alcohol metabolism, and toxicology. First, our multi-dataset integrative approach, spanning seven independent studies across different laboratories, species, and circadian disruption paradigms, enables robust cross-validation of findings and strengthens confidence in the conserved circadian regulation of ALDH2 and CYP2E1, thereby providing molecular mechanistic insight into the chronotoxicity of alcohol consumption observed in prior time-of-day studies across species [55,57,58] (Figure 1, Figure 2, Figure 3, Figure 4, Figure 5 and Figure 6). Second, the mechanistic dissection using genetic clock-deficient models (*Bmal1* KO, *Clock*Δ19) under both light–dark and constant darkness conditions definitively establishes the dual regulation of these essential alcohol metabolizing genes by intrinsic circadian machinery and extrinsic zeitgeber cues—a distinction that has been characterized for general hepatic metabolism [61] but not systematically examined for alcohol-metabolizing enzymes in prior studies [36,37,38] (Figure 2 and Figue S2). Third, the translational bridge from mouse models through human tissues to shift workers validates cross-species ALDH2 circadian regulation, as SD and HFD disrupt hepatic ALDH2 rhythms in mice (Figure 3 and Figure 4) with parallel blunting observed in PBMCs from human shift workers (Figure 6), providing molecular evidence for the exacerbated alcohol-induced liver–gut injury increasingly reported under genetic clock disruption, light–dark phase shifts, shift work, and mistimed feeding [42,45,47]. Fourth, our identification of HNF4α as a potential mediator of ALDH2 circadian vulnerability in the liver through parallel disruption patterns across multiple perturbations (sleep deprivation, high-fat diet) generates a testable mechanistic hypothesis for future experimental validation [71,103] (Figure 3 and Figure 4). Finally, the demonstrated gene-specific vulnerability hierarchy (ALDH2 >> CYP2E1/ADH1/ALDH1B1) has direct therapeutic implications: ALDH2’s unique sensitivity to circadian disruption creates temporal “high-risk windows” when acetaldehyde clearance capacity is compromised. This suggests that chronotherapy interventions could mitigate toxicity in circadian-disrupted populations, including (1) behavioral strategies such as strategic timing of alcohol consumption and circadian-aligned lighting/feeding schedules, and (2) targeted ALDH2 activation through nutritional (e.g., sulforaphane [104]) or pharmacological (e.g., Alda-1 [105]) approaches administered during vulnerable phases, thereby establishing precision medicine frameworks for alcohol-related disorders.

## 4. Materials and Methods

### 4.1. Data Sources and Acquisition 

Dataset Selection Strategy: We conducted a systematic search of the NCBI Gene Expression Omnibus (GEO) database (https://www.ncbi.nlm.nih.gov/geo/; accessed on 1 October 2025) and CircaDB (http://circadb.hogeneschlab.org/about; accessed on 1 October 2025) for circadian transcriptome studies meeting the following inclusion criteria: (1) whole-genome RNA-sequencing (RNA-seq) data from mouse liver or human tissues, (2) time-course sampling at intervals of ≤4 h across ≥24 h, (3) controlled light–dark conditions or documented circadian disruption paradigms, and (4) available raw counts or normalized expression values. Based on these criteria, we selected seven datasets representing distinct experimental contexts: baseline circadian profiling (GSE73554), genetic clock disruption (GSE70499), acute sleep deprivation (GSE262410), chronic high-fat diet feeding (GSE218932, GSE52333), human multi-tissue profiling (CircaDB), and occupational shift work (GSE122541). This multi-dataset integration strategy enables cross-validation of findings, mechanistic dissection of clock-dependent versus clock-independent regulation, and assessment of translational relevance from rodents to humans.

In this study, circadian disruption paradigms were operationally defined as follows:

(1) Genetic Circadian Disruption (GSE73554): Loss of molecular clock function through genetic deletion (*Bmal1* KO) or dominant-negative mutation (*ClockΔ19*) of core circadian transcription factors. Circadian disruption was confirmed by complete loss of rhythmicity (JTK_Cycle *p* > 0.05) in canonical clock genes (*Per1, Per2, Cry1, Cry2, Nr1d1, Nr1d2*) in constant darkness conditions [50].

(2) Acute Environmental Disruption: Sleep Deprivation (GSE262410): Six hours of enforced wakefulness during the normal rest phase (ZT0-ZT6, light phase) via gentle handling, preventing consolidated sleep while maintaining light–dark cycles. This paradigm disrupts the normal sleep–wake cycle without eliminating photic zeitgeber cues, modeling acute circadian misalignment similar to shift work or social jetlag [13]. Disruption severity was quantified by loss of rhythmicity and phase shifts in core clock genes and metabolic transcripts.

(3) Chronic Metabolic Disruption—High-Fat Diet (GSE218932, GSE52333): Three weeks (GSE218932) or ten weeks (GSE52333) of a Western-style high-fat diet (60% kcal from fat) feeding initiated during early postnatal or adolescent development. This nutritional challenge induces metabolic stress, obesity, and insulin resistance, which are known to reprogram hepatic circadian transcriptomes [14,15]. Disruption was assessed by alterations in the period, phase, and amplitude of rhythmic genes compared to low-fat diet controls.

(4) Occupational Circadian Misalignment—Night Shift Work (GSE122541): Chronic exposure to inverted work-rest schedules in rotating night-shift hospital nurses, with blood sampling conducted on a day off under controlled conditions (dim light, scheduled meals). This real-world circadian disruption involves sustained misalignment between the endogenous circadian clock and behavioral/environmental cycles [17] (PMID: 29735673). Disruption was quantified by changes in the period, phase, and amplitude of clock-controlled genes in peripheral blood mononuclear cells.

All datasets analyzed in this study were obtained from previously published sources, as indicated by their GEO accession numbers and corresponding references. No new animal experiments or human subject recruitments were conducted. Instead, we performed original integrative analyses across existing circadian transcriptome datasets, focusing specifically on oxidative alcohol-metabolizing enzymes, which were not the primary emphasis of the original studies. Our analysis pipeline, including data reprocessing, rhythm detection, and cross-condition comparisons, was designed to extract novel insights into the temporal regulation of these enzymes beyond the scope of the original publications.

*Mouse Circadian Transcriptome Datasets*: For baseline circadian expression analysis (Figure 1), we analyzed dataset GSE73554, which contains liver samples from male C57BL/6J mice (10–14 weeks of age) collected every 2 h over 94 h under standard 12 h light/12 h dark (LD) conditions with ad libitum feeding. For the *Bmal1* knockout experiments (Figure 2), we utilized dataset GSE70499, which includes two experimental conditions: (1) conventional *Bmal1* KO mice and wild-type littermates (6.4–13.9 weeks of age) housed under 12 h LD conditions, with samples collected every 4 h for 20 h, and (2) postnatal tamoxifen-induced *Bmal1* knockout (iKO) mice and wild-type controls maintained in constant darkness (DD), with samples collected every 4 h in circadian time (CT). For the sleep deprivation studies (Figure 3 and Figure S3), we analyzed dataset GSE262410, which contains liver samples from male C57BL/6J mice (10–12 weeks of age) subjected to acute sleep deprivation. Mice were sleep-deprived for 6 h (ZT0–ZT6) on Day 1 during the light phase using gentle handling. Liver samples were collected every 4 h before (Day 0–1), during (Day 1–2), and after (Day 2–3) sleep deprivation under 12 h LD conditions with ad libitum feeding. Each time point included 3–4 biological replicates, with eight ZT0 controls collected from two animal batches. For the Western-style high-fat diet experiments (Figure 4 and Figure S4), we used two independent datasets. The primary analysis (Figure 4) utilized dataset GSE218932, which contains liver samples from 3-week-old female mice fed either a standard low-fat diet (LF) or a Western-style high-fat diet (HFD; 60% kcal from fat) for 3 weeks prior to tissue collection. Samples were collected every 4 h (ZT0, 4, 8, 12, 16, 20, and 24) over a 24 h period under 12 h LD conditions (*n* = 3 per time point for each diet). For validation (Figure S4), we analyzed dataset GSE52333 from 6-week-old male mice fed normal chow or a Western-style HFD (60% kcal from fat) for 10 weeks, with samples collected every 4 h (ZT0, 4, 8, 12, 16, 20, and 24) over 24 h under 12 h LD conditions (*n* = 3 per time point for each diet). Additional multi-tissue cycling data for mouse tissues (Figure S1) and *Clock* mutant mice (Figure S2) were obtained from the Circadian Expression Profiles Database (CircaDB; http://circadb.hogeneschlab.org/mouse, 1 October 2025), which provides pre-analyzed rhythmic gene expression data using the JTK_Cycle algorithm with a *p*-value cutoff of 0.05.

Human Circadian Transcriptome Datasets: (1) Multi-Tissue Circadian Profiling (CircaDB): For human tissue-specific circadian expression patterns (Figure 5), we utilized data from CircaDB (http://circadb.hogeneschlab.org/human, 1 October 2025; [9,49]), which includes circadian transcriptome profiles derived from the GTEx (Genotype-Tissue Expression) project [106]. Tissues were collected via rapid autopsy (<24 h postmortem) from donors aged 21–70 years with a BMI of 18.5–35 kg/m^2^, excluding individuals with metastatic cancer, recent chemotherapy or radiation, or known shift work history where available [9]. Due to the cross-sectional nature of GTEx sampling, circadian phase was reconstructed using CYCLOPS (CYCLic Ordering by Periodic Structure), which infers temporal ordering based on intrinsic oscillations of core clock genes (*BMAL1, CLOCK, PER1–3, CRY1–2, NR1D1–2*) [107]. RNA-seq data (~50 million aligned reads per sample) were analyzed using cosinor regression on the top 15,000 expressed genes per tissue, applying the model: Expression = Mesor + Amplitude × cos (Phase − Acrophase). Rhythmic genes were defined using stringent thresholds: FDR-adjusted *p*-value < 0.05, relative amplitude (rAMP) ≥ 0.1 (≥10% oscillation), and R^2^ ≥ 0.1 (≥10% variance explained). Circadian phase is represented on a 24 h scale where phase 0 (2π) corresponds to biological night onset (~21:00–22:00), π/2 to mid-sleep (~03:00–04:00), π to biological day onset (~09:00–10:00), and 3π/2 to mid-wake (~15:00–16:00). We extracted expression data for *ADH1, CYP2E1, ALDH2,* and *ALDH1B1* from CircaDB across liver, lung, heart (atrial appendage), colon, and arterial tissues (aorta, coronary, tibial), and identified rhythmicity using thresholds of FDR < 0.05 and relative amplitude (rAMP) > 0.1. (2) Human shift-worker data: For the nurse shift-work dataset (GSE122541) (Figure 6), we referenced the original study by Archer et al. [18], which investigated circadian rhythms in peripheral blood mononuclear cells (PBMCs) from shift-working nurses. The study included 9 female night-shift nurses (permanent night duty) and 8 female day-shift nurses, aged ~30–35 years. Each participant completed a ~9-day protocol, during which night-shift workers performed at least three consecutive 12 h overnight shifts and day-shift workers completed corresponding daytime shifts. On the subsequent day off (sampling day), all participants were kept under controlled laboratory conditions, a constant dim red-light environment with isocaloric, time-matched meals, to eliminate external zeitgeber influence. PBMCs were collected every 3 h over a 24 h period to capture endogenous circadian expression. Sampling after the shift-work block ensured the night-shift group was in a misaligned circadian state. Indeed, physiological markers such as dim-light melatonin onset and core body temperature minimum were significantly delayed or desynchronized in night-shift workers, confirming circadian disruption [18]. We reanalyzed the publicly available transcriptome data from this experiment (GSE122541), treating the day-shift and night-shift groups separately, to assess how chronic circadian misalignment alters the rhythmic expression of alcohol-metabolizing enzymes compared to aligned controls.

RNA-Sequencing Data Extraction and Processing: For each RNA-seq time course dataset, we downloaded the available gene expression matrices, which were either raw count files (from GEO Series Matrix or supplementary text/CSV files) or processed normalized data (e.g., FPKM, TPM, or study-provided normalized counts). Raw count files were imported into Microsoft Excel and, when necessary, normalized to transcripts per million (TPM) or reads per kilobase million (RPKM) to ensure comparability across samples. For datasets where only processed data were provided, the normalized values were used directly without additional transformation. All expression tables were converted into Excel format and organized into separate worksheets containing sample metadata (genotype, treatment condition, ZT or CT, and replicate information) as well as expression values for our genes of interest (*Adh1, Cyp2e1, Aldh2, Aldh1b1, Hnf4a,* and *Ppard*) and core clock genes (*Bmal1, Clock, Per1, Per2, Per3, Cry1, Cry2, Nr1d1, Nr1d2, Rora,* and *Rorb*). For each time point, mean expression values and standard error of the mean (SEM) were calculated. All datasets were chronologically arranged by ZT (for LD conditions) or CT (for DD conditions) to facilitate subsequent circadian analyses.

### 4.2. Circadian Rhythm Analysis

BioDare2 Analysis: Circadian rhythm parameters for gene expression (period, amplitude, and phase) (Figure 1 and Figure 6) were estimated using the BioDare2 online platform (https://biodare2.ed.ac.uk; accessed to 20 November 2025). Time-course expression data (with time in hours and expression values for each gene or averaged replicates) were uploaded in CSV format. We applied BioDare2’s Fast Fourier Transform–Nonlinear Least Squares (FFT-NLLS) algorithm to detect rhythmic patterns in the data. The analysis settings were a period search range of 20–28 h, linear baseline detrending, and normalization to relative amplitude units. BioDare2 generated best-fit sinusoidal (cosine) curves for each time series and provided estimates of the circadian period, phase, and amplitude for each gene. For visualization, these fitted curves were overlaid on the raw data points. Phase values are reported in units of ZT (or CT for constant conditions), where ZT0 (or CT0) corresponds to the time of lights-on (subjective dawn in DD conditions).

MetaCycle/JTK_Cycle Analysis: We also assessed circadian rhythmicity using the MetaCycle R package (v1.2.0), which integrates multiple algorithms for rhythm detection (JTK_CYCLE, Lomb-Scargle, and ARSER) [108]. The MetaCycle output included, for each gene, a JTK_CYCLE *p*-value (*p*^Cycle^) indicating the statistical significance of 24 h rhythmicity (with *p* < 0.05 considered significant), the estimated period (in hours), the phase (peak expression time, in ZT or CT), and the amplitude (relative oscillation magnitude). For experiments with multiple conditions or genotypes (e.g., WT vs. KO, low-fat vs. high-fat diet), we performed separate rhythm analyses for each subgroup to compare their circadian parameters.

Phase Vector Analysis: For the high-fat diet experiments (Figure 4D–F), phase relationships were visualized using circular phase plots. Phase values (peak expression times) from the MetaCycle/JTK_Cycle analysis were converted to radians (0 to 2π corresponding to 0 to 24 h) and plotted on circular coordinate systems with ZT0 positioned at 0° (12 o’clock position) and time progressing clockwise. Vector length represents the strength of rhythmicity, with longer vectors indicating more robust rhythms.

### 4.3. CircaDB Data Retrieval

For circadian expression data from CircaDB, we accessed the online database interface and searched for genes of interest across multiple mouse and human tissues. CircaDB provides JTK_Cycle-based rhythm detection with FDR correction for multiple testing. For the mouse data, we used the default threshold of JTK_Cycle *p* < 0.05. For the human tissue data, we applied more stringent criteria of FDR < 0.05 and relative amplitude (rAMP) > 0.1 to identify robustly rhythmic genes. Circadian expression traces and fitted curves were downloaded directly from CircaDB as image files. For genes meeting rhythmicity criteria, we extracted phase, period, and amplitude parameters from the database output tables.

### 4.4. Data Visualization

Expression Profile Plotting: Time-course expression profiles were plotted using GraphPad Prism (version 10). For each gene, mean normalized expression values ± SEM were plotted against ZT or CT. Light–dark cycles are indicated by alternating white (light phase, ZT0–12) and black (dark phase, ZT12–24) shading in the background of plots. For datasets with rhythmic patterns, fitted cosine curves from the BioDare2 analysis were overlaid on the raw data points to visualize the circadian oscillation patterns. Expression data were presented as relative mRNA expression (normalized to mean expression across all time points) or absolute normalized counts/FPKM values, depending on the original dataset format.

Heatmap Generation: For the shift-worker data (Figure 6C), heatmaps were generated using the BioDare2 online platform after normalizing expression values to each gene’s mean and calculating row-scaled *z*-scores. Time points are arranged horizontally and genes vertically, with color scales ranging from blue (low expression) to red (high expression), and white indicating mean expression.

### 4.5. Statistical Analysis

All data are presented as mean ± SEM unless otherwise noted. Circadian rhythmicity was assessed using MetaCycle/JTK_Cycle *p*-values (*p*^Cycle^), with *p*^Cycle^ < 0.05 indicating significant 24 h rhythms. Data analysis and visualization were performed using GraphPad Prism 10, R (v4.0.0) with MetaCycle 1.2.0 and pheatmap 1.0.12, and Microsoft Excel 2024 for data organization. Circadian analyses were conducted using BioDare2 and the MetaCycle R package.

## 5. Conclusions

Our comprehensive circadian transcriptome analysis establishes that alcohol-metabolizing enzymes display gene- and tissue-specific circadian oscillations, with *ALDH2* showing both the strongest rhythmicity and the greatest sensitivity to circadian disruption across species. The BMAL1-independent but light–dark cycle-dependent rhythmicity of these enzymes reveals the capacity for systemic circadian signals to impose metabolic coordination, even in the absence of local clock function. Most importantly, the exceptional vulnerability of *ALDH2* to SD, a Western-style HFD, and shift work suggests that circadian disruption may specifically compromise acetaldehyde detoxification, creating potential temporal windows of increased toxicity upon alcohol drinking. These findings advance our understanding of the bidirectional interaction between circadian regulation and alcohol metabolism by (1) providing a potential molecular mechanism underlying increased vulnerability to alcohol-induced toxicity and organ damage in circadian-disrupted rodents and humans; (2) highlighting the importance of environmental and behavioral factors, including timing of alcohol intake, diet, sleep quality, shift work, jet lag, and social jet lag, that influence circadian homeostasis and alcohol detoxification; and (3) establishing a foundation for developing chronotherapeutic strategies to optimize alcohol detoxification efficiency in populations experiencing circadian disruption. Looking forward, these findings have important implications for public health and precision medicine, suggesting that monitoring circadian health, through assessments of sleep–wake patterns, occupational history, or wearable rhythm tracking [109], could inform individualized recommendations for alcohol consumption timing and quantity, particularly for populations with irregular schedules such as shift workers and those at risk of alcohol use disorders.

## Data Availability

All data analyzed in this study are publicly available through the Gene Expression Omnibus (GEO) database under accession numbers GSE73554, GSE70499, GSE262410, GSE218932, GSE52333, and GSE122542. Circadian expression data from mouse and human tissues were obtained from the CircaDB database (http://circadb.hogeneschlab.org, 1 October 2025).

## Supplementary Materials

The following supporting information can be downloaded at: https://www.mdpi.com/article/10.3390/ijms27042041/s1.
