# Supplementary material for: Diurnal Regulation and Gene-Specific Vulnerability of Oxidative Alcohol-Metabolizing Enzymes to Circadian Disruption
Source: Int J Mol Sci. 2026 Feb 22;27(4):2041. doi: 10.3390/ijms27042041 (PMC12940399; doi:10.3390/ijms27042041)
Supplement: Supplementary file 1 [file ijms-27-02041-s001.zip › ijms-4110007-supplementary.pdf]

Supplementary Figures

Figure S1

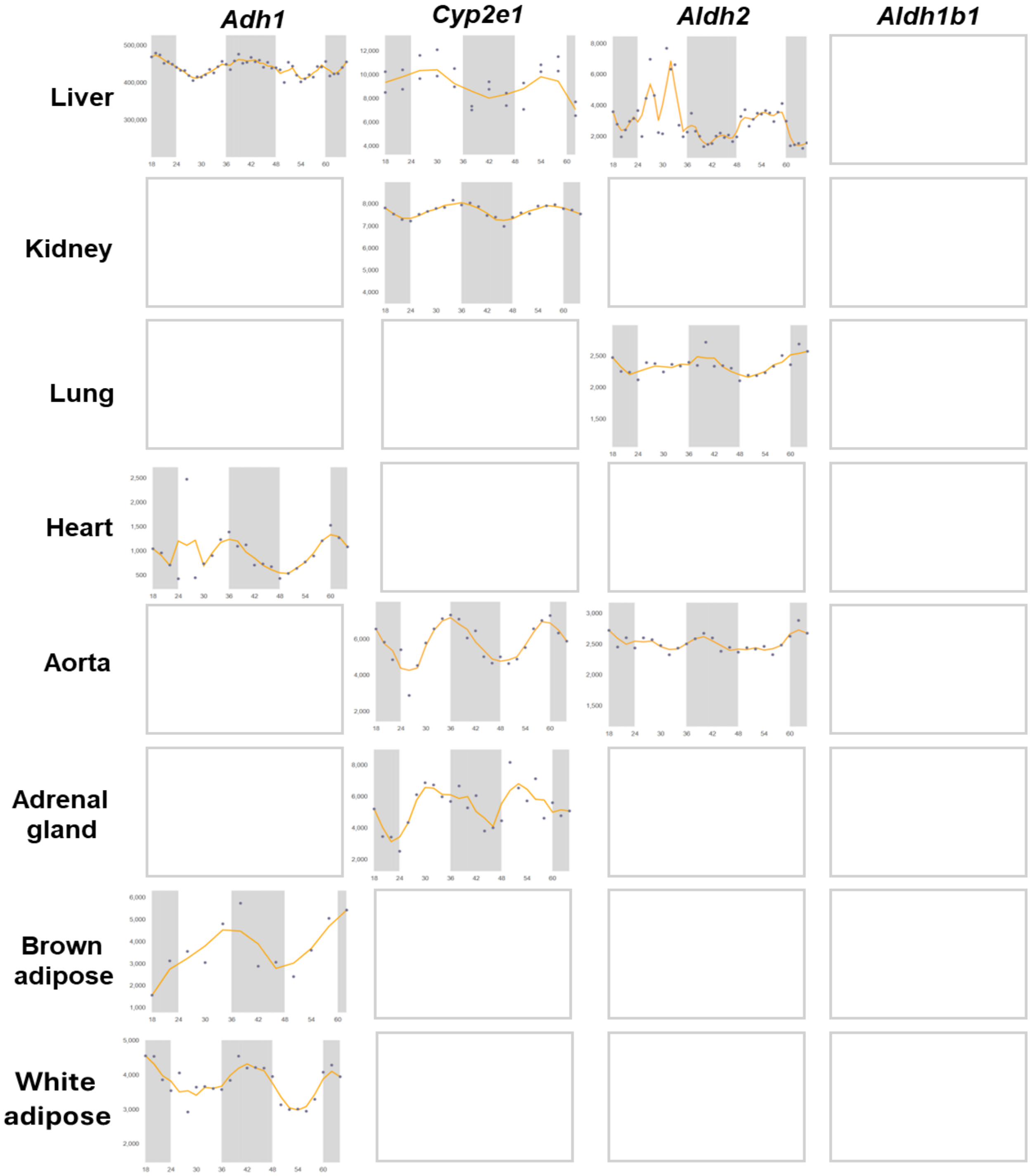

Figure S1. Gene- and tissue-specific diurnal oscillations of alcohol-metabolizing enzymes in mouse tissues. Diurnal expression profiles of *Adh1*, *Cyp2e1*, *Aldh2*, and *Aldh1b1* across the indicated mouse tissues. Each dot represents normalized gene expression from an individual sample plotted against its in-

ferred circadian phase. The orange line represents the fitted harmonic regression curve modeling rhythmic expression across the 24-h cycle. Data analyzed were obtained from the Circadian Expression Profiles Database (CircaDB; http://circadb.hogeneschlab.org/mouse). Rhythmic gene expression was identified using a JTK_Cycle *p*-value cutoff of 0.05. Expression traces that did not meet the JTK_Cycle significance threshold ($p < 0.05$) are intentionally indicated with blank, gray rectangular boxes.

Figure S2

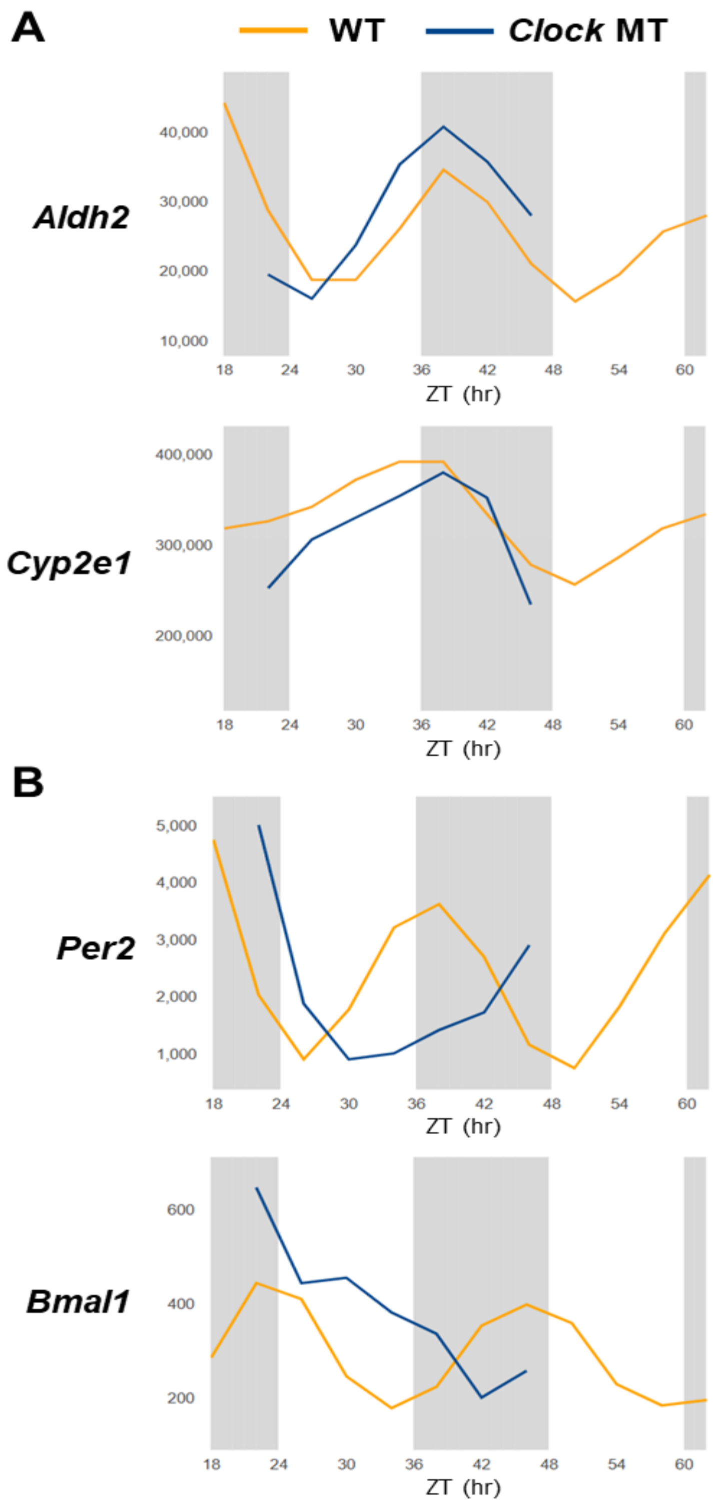

Figure S2. Light-dark cycles preserve *Aldh2* and *Cyp2e1* rhythmicity in *Clock* mutant mice. (A, B) Time-series mRNA expression profiles of alcohol-metabolizing enzymes (A: *Aldh2*, *Cyp2e1*) and core

clock genes (B: *Per2*, *Bmal1*) in liver tissues of wild-type (WT; yellow) and *Clock* Δ19 mutant (*Clock* MT; blue) mice. Rhythmic profiles were identified using a JTK_Cycle *p*-value cutoff of 0.05 on data obtained from the Circadian Expression Profiles Database (CircaDB; http://circadb.hogeneschlab.org/mouse).

Figure S3

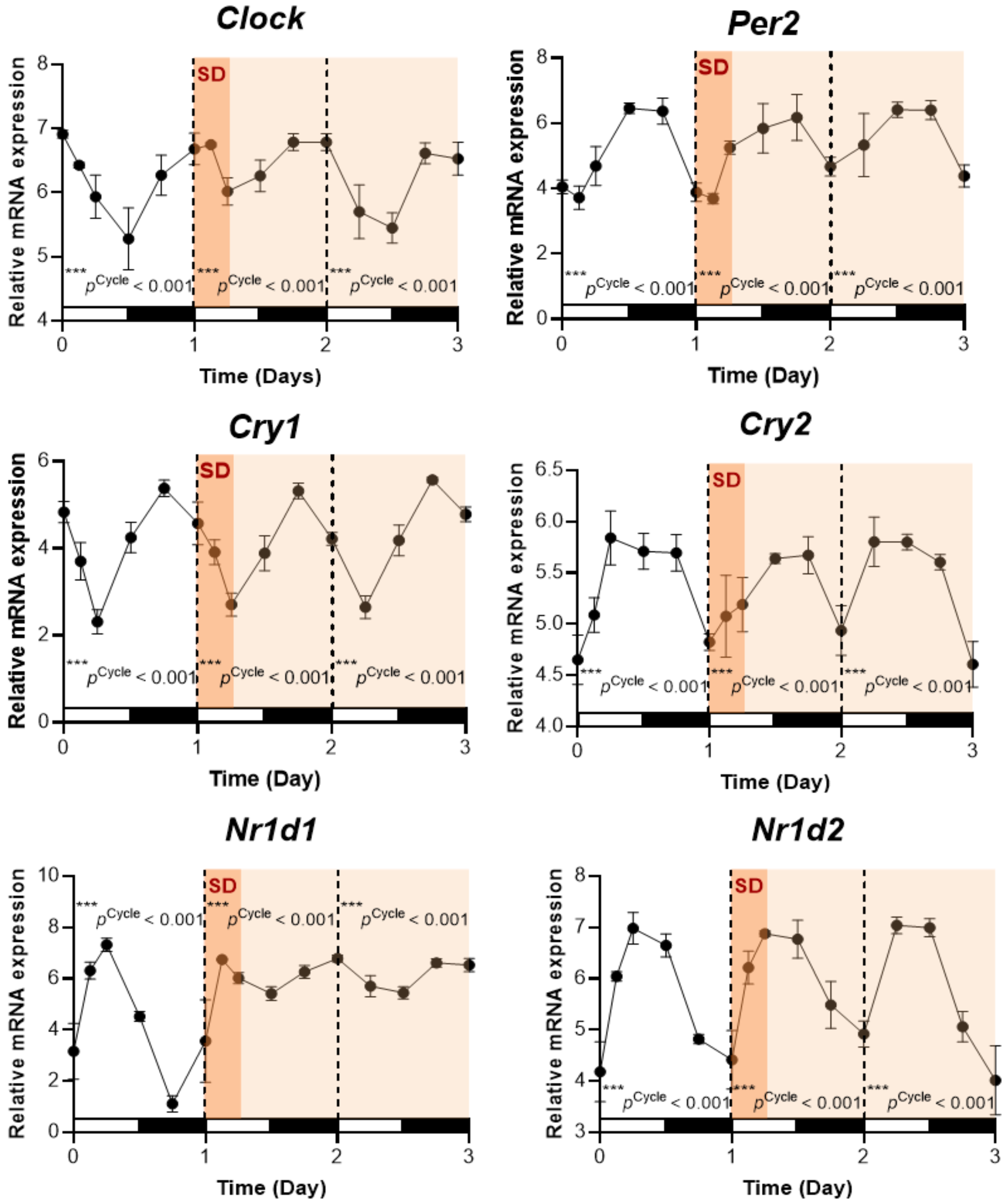

Figure S3. Acute sleep deprivation differentially alters the diurnal oscillations of core clock genes in mouse liver. Time-series mRNA expression profiles of the indicated circadian clock genes in mouse livers before (Day 0–1), during (on Day 1), and after (Days 1–3, light orange) acute sleep deprivation (SD; dark orange). Liver samples were collected every 4 hours from mice housed under 12-h light/12-h dark conditions (lights on at ZT0 and off at ZT12) with ad libitum feeding. Sleep deprivation (SD) was for 6 hours from ZT0 to ZT6 on Day 1 during the light (resting) phase. Each time point included 3–4 biological replicates, and eight ZT0 controls were collected from two animal batches.

MetaCycle/JTK_Cycle rhythm analysis results (p^{Cycle}) for each gene and day are shown on the graphs. $p^{Cycle} < 0.05$ indicates significant 24-hour rhythmicity. Data were generated from publicly available global RNA-seq datasets (GSE262410).

Figure S4

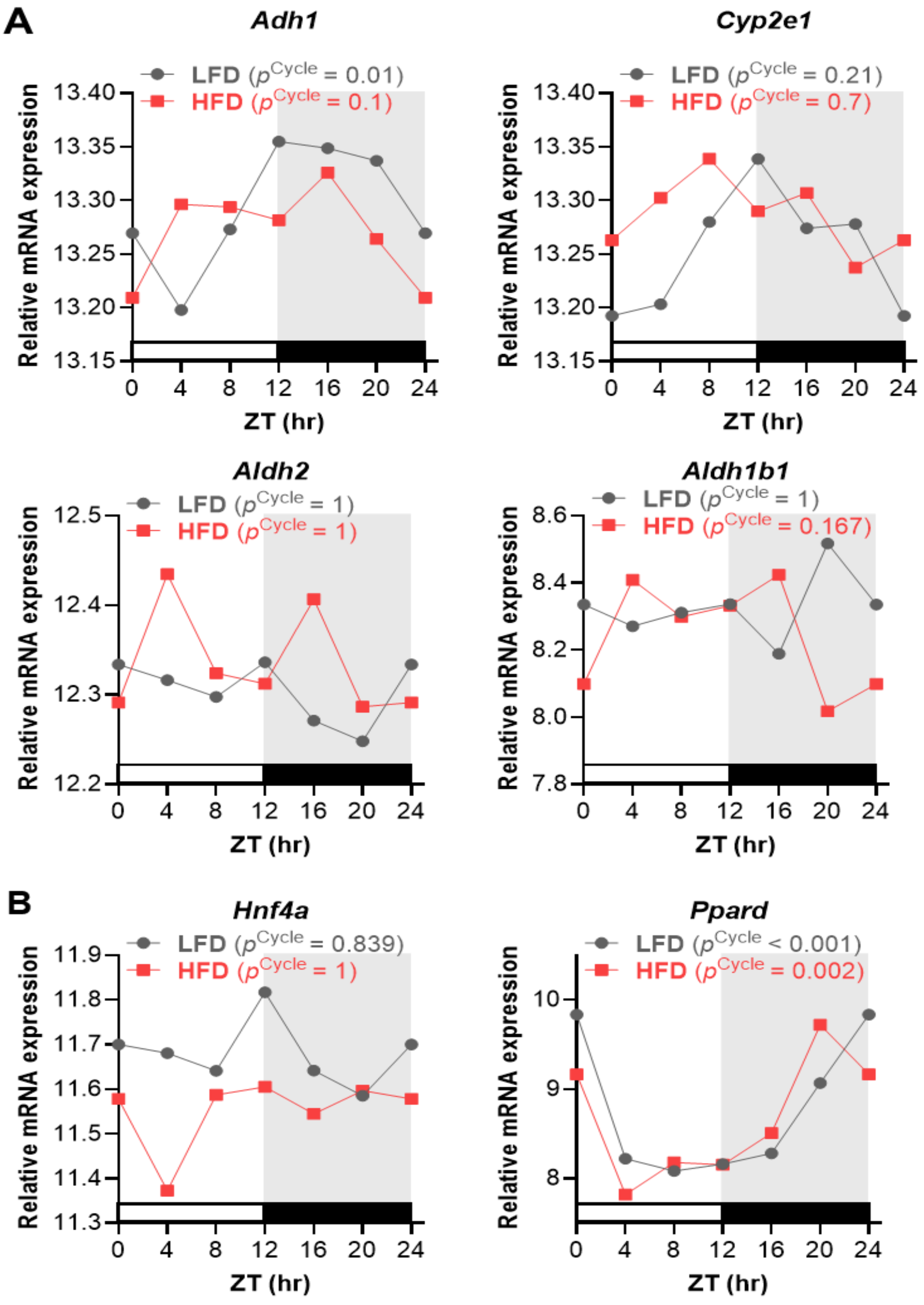
A
Adh1
LFD (p^{Cycle} = 0.01)
HFD (p^{Cycle} = 0.1)
Relative mRNA expression
13.40
13.35
13.30
13.25
13.20
13.15
0 4 8 12 16 20 24
ZT (hr)
Cyp2e1
LFD (p^{Cycle} = 0.21)
HFD (p^{Cycle} = 0.7)
Relative mRNA expression
ZT (hr)
Aldh2
LFD (p^{Cycle} = 1)
HFD (p^{Cycle} = 1)
Relative mRNA expression
12.5
12.4
12.3
12.2
ZT (hr)
Aldh1b1
LFD (p^{Cycle} = 1)
HFD (p^{Cycle} = 0.167)
Relative mRNA expression
8.6
8.4
8.2
8.0
7.8
ZT (hr)
B
Hnf4a
LFD (p^{Cycle} = 0.839)
HFD (p^{Cycle} = 1)
Relative mRNA expression
11.9
11.8
11.7
11.6
11.5
11.4
11.3
ZT (hr)
Ppard
LFD (p^{Cycle} < 0.001)
HFD (p^{Cycle} = 0.002)
Relative mRNA expression
10
9
8
ZT (hr)

Figure S4. A high-fat diet (HFD) differentially reprograms the diurnal expression of hepatic alcohol-metabolizing enzymes in mouse liver. (**A, B**) Temporal hepatic mRNA expression profiles of alcohol-metabolizing enzymes (*Adh1*, *Cyp2e1*, *Aldh2*, *Aldh1b1*) and transcriptional regulators (*Hnf4a*, *Ppard*) in male mice with diet intervention initiated at 6 weeks of age and fed a low-fat control diet (LFD; grey) or a Western-style high-fat diet (HFD; 60% kcal from fat; red) for 10 weeks, with samples collected every 4 h across a 24-h light–dark cycle (n = 3 per ZT per diet). Data represent mean values (n = 3 per ZT for each diet). White bars denote the light phase and black bars denote the dark phase. JTK_Cycle p-values (p^{Cycle}) for rhythmicity under LFD and HFD are shown above each panel. $p^{Cycle} < 0.05$ indicates significant 24-hour rhythmicity. Data were obtained from global RNA-seq datasets (GSE52333).
